# Association between secondhand smoke exposure and early eruption of deciduous teeth: A cross-sectional study

**DOI:** 10.18332/tid/84892

**Published:** 2018-02-21

**Authors:** Takashi Hanioka, Miki Ojima, Keiko Tanaka, Nao Taniguchi, Kaoru Shimada, Takeshi Watanabe

**Affiliations:** 1 Department of Preventive and Public Health Dentistry, Fukuoka Dental College, Fukuoka, Japan; 2 Department of Oral Health Sciences, Faculty of Nursing and Health Care, BAIKA Women’s University, Osaka, Japan; 3 Department of Epidemiology and Preventive Medicine, Ehime University Graduate School of Medicine, Ehime, Japan; 4 Department of Nursing, Faculty of Nursing, Fukuoka Nursing College, Fukuoka, Japan

**Keywords:** tooth eruption, deciduous tooth, secondhand smoke, maternal smoking, household smoking

## Abstract

**INTRODUCTION:**

Secondhand smoke (SHS) exposure is a risk factor for early childhood caries. Here we examined the association between SHS exposure and early tooth eruption (ETE) to clarify the additional etiology of an increased chance of contact between the tooth’s surface and acid produced by fermenting oral bacteria.

**METHODS:**

Data of 388 child–mother pairs who attended health checkups at public health centers were assessed for children aged ≥18 months. SHS exposure was reported as maternal smoking during pregnancy and household smoking after birth. Associations between SHS exposure and ETE (≥3 canines in the oral cavity) were tested using multivariable analyses of the dose-response relationship. Subgroup and sensitivity analyses were performed for birth-weight subgroups and SHS exposure variables, respectively.

**RESULTS:**

ETE prevalence was 65.5%, 68.1%, and 76.9% in the no, medium-dose (ceased partway and sometimes), and highest-dose (every day) exposure groups, respectively, during pregnancy, and 61.5%, 75.0%, and 75.5%, respectively, after birth. The association between the highest dose exposure during pregnancy and ETE was not significant (OR=1.42, 95% CI: 0.34–5.96, p=0.631), whereas that between highest dose exposure after birth and ETE was significant (OR=2.13, 95% CI: 1.06–4.31, p=0.034); this association was distinct in the subgroup of children with smaller birth weights (<3000 g) (OR=3.19, 95% CI: 1.08–9.44, p=0.036). The dose-response relationship was consistently significant for exposure after birth (p<0.05). The sensitivity analysis that employed no SHS exposure, as a reference, revealed that exposure after birth but no exposure during pregnancy was significantly associated with ETE (OR=2.29, 95% CI: 1.19–4.40, p=0.013). However, the association between exposure during pregnancy and ETE was consistently non-significant (p>0.05).

**CONCLUSIONS:**

When controlling for variables of birth weight and exposure type, SHS exposure after birth was independently associated with the early eruption of deciduous canines. Further studies are warranted to examine the trajectory of SHS exposure after birth, ETE, and early childhood caries incidence.

**ABBREVIATIONS:**

ETE: Early tooth eruption, SHS: Secondhand smoke

## INTRODUCTION

Secondhand smoke (SHS) exposure causes significant morbidity and mortality in children^1^, and adversely influences oral health in addition to respiratory health^2,3^. Maternal smoking exposure is associated with congenital defects of the orofacial cleft^4^, whereas SHS exposure is associated with early childhood caries^4-6^. Although deciduous teeth are eventually replaced with permanent teeth, early childhood caries are a public health problem, because they are associated with toothache, mastication, orofacial development, and permanent tooth alignment, in children.

The primary etiology of dental caries is cariogenic contact between premature enamel crystals of erupting teeth and acid produced by fermenting oral bacteria harboring on a tooth’s surface^7^. Tobacco smoke exposure increases the susceptibility to acid because: calcification of the enamel matrix is inhibited^8^, enamel crystals grow prematurely^9^, and acid-producing plaques are enhanced^10,11^ on developmental defects of the tooth’s surface^12^. Early tooth eruption (ETE) may be added to the etiology of dental caries because ETE in the oral cavity could increase the likelihood of cariogenic contact^13^.

However, the association between SHS exposure during pregnancy after birth and early childhood caries remains unclear. An additive effect of prenatal maternal smoking and postnatal SHS exposure on early childhood caries was observed^14^. The hazard ratio for early childhood caries between cases of maternal smoking during pregnancy and those of no smoking in the family was 1.10 in a cohort of 12 729 children, while the associated risk from tobacco smoke exposure after birth increased by 2.14^15^. Similarly, studies that assessed the association between the timing of tooth eruption and SHS exposure, including maternal smoking, have reported inconsistent findings^16-18^, with one study reporting no significant effect^17^. The first deciduous tooth eruption occurred approximately 1 week earlier in children who were exposed to maternal smoking during pregnancy compared with those who were not exposed^16^, and deciduous tooth eruption negatively correlated with maternal smoking exposure during pregnancy^18^. Furthermore, low birth weight of <2500 g is associated with delayed tooth eruption^19^, and premature birth is associated with SHS exposure^1^.

Understanding the association between SHS exposure and early childhood caries with regard to the timing of exposure and interaction with birth weight is difficult. Thus, clarifying the potential association between SHS exposure and ETE may help to explaining the effect of such an exposure on increased susceptibility to early childhood caries. Here, we assessed the association between SHS exposure and ETE of deciduous teeth with regard to birth weight and timing of exposure.

## METHODS

### Study population

In Japan, periodic health checkups for infants at public health centers are required, by law, to include oral health examinations at the age of 18–23 months; but most children undergo checkups at the age of 18 or 19 months. On the basis of the smoking rate of mothers of 18-month-old children and the expected rate of dropout, a sample size of 450 was considered to be appropriate. The survey was performed from March to September 2014 at public health centers in three cities in Shizuoka Prefecture. Permission for conducting this present study was obtained from the local government of each city. The study protocol was approved by the Ethics Committee of the Fukuoka Dental College (Ethics Approval No. 224).

### Data collection

The mailed notices regarding health checkups of 485 children included a questionnaire and an informed consent form. Addresses were obtained from the basic resident register according to the date of birth. Written informed consent was obtained from the guardians of all children. Health records and questionnaires that were completed by the children’s guardians were used to assess the association between SHS exposure and ETE. Other data of the enrolled children, such as existing teeth, were transcribed from the health records after oral examination. The dentists who usually examined children at the public health centers were notified of the study objective in advance but were blinded to the children’s exposure status to reduce the information bias of the examiners.

### Outcome measure of ETE

In the study population, ≥50% of the children had 16 teeth, and almost no children had four second-deciduous molars. In general, except for the second deciduous molars, canines are the last teeth to emerge in the oral cavity of children at the age of 18–23 months. Therefore, the number of canines was used as a measure of ETE. In this study, the presence of three or four canines in the oral cavity was defined as ETE. Furthermore, emergence of at least one part of the tooth structure in the oral cavity was defined as an existing tooth, and fused teeth were recorded as two independent teeth. Because this definition of an existing tooth is universal in Japan, no calibration among the dentists was performed. The ETE variable was dichotomized as a dependent variable for statistical analyses.

### Assessment of SHS exposure

Data concerning each child’s SHS exposure were obtained on the basis of two questions that assessed maternal smoking during pregnancy and household smoking around the child after birth. In addition, data regarding the use of other tobacco products and exposure to thirdhand smoke were excluded because such products and concepts were not popular in Japan at the time of survey. For each question, the mother selected one response from the following four categories: no, sometimes, every day, and ceased partway. Because the number of children in the ‘sometimes’ and ‘ceased partway’ categories of SHS exposure was small for statistical analyses, these categories were combined into a single exposure category that comprised the medium-dose exposure category.

### Potential confounders

Gender (male or female), location of residence (city A, B, or C), birth order (first or other than first), and parents’ education level (no more than high school or college level or above; the higher level was recorded in cases where the parents’ education level differed), and annual income (<3, 3–4.9, or ≥5 million yen) were included in the statistical model as covariates of categorical variables, and the first category of each variable was used as reference. According to the monthly average in March 2014, one million yen was equivalent to 9778 US dollars. Age and birth weight were included in the model as covariates of continuous variables. Data regarding the variables of age, birth order, and birth weight of children living in city A were transcribed from health records, whereas those for children living in cities B and C were obtained from the questionnaire.

### Statistical analysis

Datasets with no missing values were used for the multivariable analyses. Chi-squared test and one-way ANOVA were used for bivariate analyses. Multivariable logistic regression analyses were performed to independently test the association between SHS exposure and ETE, when all independent variables were included. Children were divided into two subgroups according to birth weight (<3000 g and ≥3000 g). Each group was subjected to a subgroup analysis wherein birth weight was included as a continuous variable. Trends were analyzed by incorporating SHS exposure variables as ordinal variables. For sensitivity analyses, children not exposed to maternal smoking during pregnancy or household smoking after birth were used as reference. For the maternal smoking question, the no exposure group was divided into ‘no exposure’ and ‘exposure to household smoking only’ groups. For the household smoking question, the no exposure group was divided into ‘no exposure’ and ‘exposure to maternal smoking during pregnancy only’ groups. These analyses were conducted using statistical software (IBM SPSS 20.0; IBM Corp., New York, NY, USA), with the significance level set at 5%.

## RESULTS

### Characteristics regarding SHS exposure

Data of 430 children (88.7% of invited children) were collected. Finally, datasets of 388 (80.0%) pairs without values that were either missing or incorrect were used for the analyses. The distribution of children according to the questions regarding SHS exposure is summarized in a contingency table (Table 1). Most children (63.4%) were not exposed to SHS. Maternal smoking exposure during pregnancy and household smoking exposure after birth were documented in 15.5% and 31.7% of children, respectively.

**Table 1:** Contingency table of SHS exposure variables

		*Level of maternal smoking exposure during pregnancy*			
		*No exposure*	*Medium dose^a^*	*Highest dose^b^*	*Total*
Level of household smoking exposure after birth	No	246 (63.4%)	17 (4.4%)	2 (0.5%)	265 (68.3%)
	Medium dose^a^	36 (9.3%)	15 (3.9 %)	1 (0.3%)	52 (13.4%)
	Highest dose^b^	46 (11.9%)	15 (3.9%)	10 (2.6%)	71 (18.3%)
	Total	328 (84.5%)	47 (12.1%)	13 (3.4%)	388 (100%)

The association between two measures of exposure was significant at p< 0.001 (χ^2^ test). Two cells (22.2%) had an expected frequency of <5. Category of questionnaire:

a ‘sometimes and ceased partway’ and

b ‘every day’.

### Characteristics of children according to SHS exposure and ETE

Table 2 shows the distribution and characteristics of children according to SHS exposure levels that were stratified based on potential confounders. For the categorical variables, significant negative associations were observed between both the types of SHS exposure and parents’ education level and annual income, whereas gender, location of residence, and birth order were not significantly associated with SHS exposure. Among the continuous variables, negative association between birth weight and maternal smoking during pregnancy was significant; whereas associations between birth weight and household smoking after birth, or between age and SHS exposure, were not significant.

**Table 2:** Distribution and characteristics of children according to two measures of SHS exposure stratified based on potential confounders

*SHS exposure levels*							
*Variables and categories*	*Maternal smoking during pregnancy*			*Household smoking after birth*			*Total*
	No exposure	Medium dose^a^	Highest dose^b^	No exposure	Medium dose^a^	Highest dose^b^	
*Number (%) for categorical variables*							
**Gender**							
Male	165(83.3)	26(13.1)	7(3.5)	133(67.2)	28(14.1)	37(18.7)	198(100)
Female	163(85.8)	21(11.1)	6(3.2)	132(69.5)	24(12.6)	34(17.9)	190(100)
	p=0.796			p=0.872			
**Location of residence**							
A city	55(80.9)	10(14.7)	3(4.4)	49(72.1)	5(7.4)	14(20.6)	68(100)
B city	178(81.7)	30(13.8)	10(4.6)	138(63.3)	35(16.1)	45(20.6)	218(100)
C city	95(93.1)	7(6.9)	0(0)	78(76.5)	12(11.8)	12(11.8)	102(100)
	p=0.060^c^			p=0.079			
**Birth order**							
First	151(83.9)	21(11.7)	8(4.4)	125(69.4)	21(11.7)	34(18.9)	180(100)
≥Second	177(85.1)	26(12.5)	5(2.4)	140(67.3)	31(14.9)	37(17.8)	208(100)
	p=0.530			p=0.643			
**Highest education of parents**							
≤High school	85(69.7)	28(23.0)	9(7.4)	52(42.6)	29(23.8)	41(33.6)	122(100)
≥College	243(91.4)	19(7.1)	4(1.5)	213(80.1)	23(8.6)	30(11.3)	266(100)
	p<0.001^d^			p<0.001			
**Annual income of parents, million yen**							
<3	38(69.1)	14(25.5)	3(5.5)	27(49.1)	13(23.6)	15(27.3)	55(100)
3–4.9	166(86.5)	18(9.4)	8(4.2)	123(64.1)	28(14.6)	41(21.4)	192(100)
≥5	124(87.9)	15(10.6)	2(1.4)	115(81.6)	11(7.8)	15(10.6)	141(100)
	p=0.007^c^			p<0.001			
**Total**	328(84.5)	47(12.1)	13(3.4)	265(68.3)	52(13.4)	71(18.3)	388(100)
*Mean ± standard deviation for continuous variables*							
**Age, months**	18.7	18.6	18.5	18.6	18.8	18.7	18.7
	± 0.9	± 0.7	± 0.5	± 0.8	± 0.9	± 0.8	± 0.8
	p=0.758			p=0.230			
**Birth weight, g**	2983.9	3026.1	2707.5	2959.7	3075.4	2984.3	2979.7
	± 409.1	± 348.2	± 371.3	± 402.9	± 417.1	± 391.3	± 403.6
	p=0.037			p=0.167			

Category of questionnaire:

a ‘sometimes and ceased partway’ and

b ‘every day’.

c Two cells (22.2%) and

d one cell (16.7%) had an expected frequency <5.

ETE prevalence was significantly associated with residence (highest prevalence for city B, p=0.011), older age (p=0.001), and higher birth weight (p=0.020), among all potential confounders (data available in appendix table 1). No significant association was observed with other variables.

### Association between SHS exposure and ETE

Overall, ETE prevalence was 66.2% (Table 3). The trend in the distribution of children with ETE according to the SHS exposure level was different for each type of SHS exposure. For maternal smoking exposure during pregnancy, ETE prevalence was 65.5% for no exposure, 68.1% for medium-dose exposure, and 76.9% for highest-dose exposure. For household smoking exposure after birth, ETE prevalence was 61.5%, 75.0%, and 77.5%, respectively. The ‘no exposure’ groups showed the lowest ETE prevalence for both types of exposure. ETE prevalence was consistently highest in the highest-dose exposure group for both types of exposure. Adjusted odds ratios (AORs) and 95% confidence intervals (CIs) of the highest-dose exposure group were 1.42, and 0.34–5.96 (p=0.631), respectively, for maternal smoking exposure during pregnancy, and 2.13, and 1.06–4.31 (p=0.034), for household smoking exposure after birth. The association between ETE and SHS exposure and the dose-response relationship were significant only for household smoking exposure after birth.

**Table 3:** Prevalence of early tooth eruption (ETE) and odds ratios (ORs) and 95% confidence intervals (CIs) of ETE according to SHS exposure level with regard to exposure type

*Exposure type*	*Exposure level*	*ETE % (n)*	*Crude*		*Adjusted*	
			*OR, 95% CI*	*p*	*OR, 95% CI*	*p*
**Maternal smoking during pregnancy**	No exposure	65.5 (215)	Reference		Reference	
	Medium dose^a^	68.1 (32)	1.12, 0.58–2.16	0.732	0.95, 0.45–1.98	0.888
	Highest dose^b^	76.9 (10)	1.75, 0.47–6.49	0.402	1.42, 0.34–5.96	0.631
			p for trend = 0.479		p for trend = 0.268	
**Household smoking after birth**	No exposure	61.5 (163)	Reference		Reference	
	Medium dose^a^	75.0 (39)	1.88, 0.96–3.69	0.067	1.71, 0.80–3.63	0.163
	Highest dose^b^	77.5 (55)	2.15, 1.17–3.96	0.014	2.13, 1.06–4.31	0.034
			p for trend = 0.004		p for trend = 0.011	
	Total	66.2 (257)				

Category of questionnaire:

a ‘sometimes and ceased partway’ and

b ‘every day’.

### Subgroup analysis

Table 4 shows ETE prevalence, and crude and AORs of ETE prevalence according to the SHS exposure level based on exposure type in children with birth weights of <3000 g. The trend in the distribution of children according to the SHS exposure level was also different for each type of SHS exposure. For the no, medium-dose, and highest-dose exposure groups, ETE prevalence was 58.1%, 69.6%, and 72.7%, respectively, for maternal smoking during pregnancy, and 54.2%, 68.2%, and 80.0%, respectively, for household smoking after birth. Compared to the AORs of the highest-dose exposure groups of all children (Table 3), that of exposed children to maternal smoking during pregnancy was lower, 0.98 vs 1.42, whereas that of exposed children to household smoking after birth was higher, 3.19 vs 2.13. The dose-response relationship was negative for maternal smoking exposure during pregnancy. Regarding household smoking exposure after birth, the association and dose-response relationship were again significant (p= 0.036 and 0.019, respectively). No significant association between SHS exposure and ETE was found in children with birth weights of ≥3000 g (data available in appendix table 2).

**Table 4:** Prevalence of early tooth eruption (ETE) and odds ratios (ORs) and 95% confidence intervals (CIs) of ETE according to SHS exposure level with regard to exposure type in children with birth weights of <3000 g

*Exposure type*	*Exposure level*	*Crude*		*Adjusted*	
		*ETE % (n/total n)*	*OR, 95% CI*	*p*	*OR, 95% CI*	*p*
**Maternal smoking during pregnancy**	No exposure	58.1 (97/167)	Reference		Reference	
	Medium dose^a^	69.6 (16/23)	1.65, 0.64–4.22	0.297	1.68, 0.54–5.25	0.375
	Highest dose^b^	72.7 (8/11)	1.92, 0.49–7.51	0.346	0.98, 0.20–4.84	0.980
			p for trend = 0.173		p for trend = 0.263	
**Household smoking after birth**	No exposure	54.2 (78/144)	Reference		Reference	
	Medium dose^a^	68.2 (15/22)	1.81, 0.70–4.71	0.222	1.29, 0.43–3.90	0.656
	Highest dose^b^	80.0 (28/35)	3.39, 1.39–8.25	0.007	3.19, 1.08–9.44	0.036
			p for trend = 0.004		p for trend = 0.019	
	Total	60.2 (121/201)				

Category of questionnaire:

a ‘sometimes and ceased partway’ and

b ‘every day’.

### Sensitivity analysis

Table 5 shows ETE prevalence, crude and AORs of ETE prevalence obtained in the sensitivity analyses of the models for maternal smoking exposure during pregnancy and/or household smoking exposure after birth. ETE prevalence in the category of exposure after birth only was the highest (79.3%) among the four exposure groups. The AOR and 95% CI were 2.29 and 1.19–4.40 (p= 0.013). ETE prevalence was higher in children in the exposure during pregnancy only (68.4 %) and both types of exposure (70.7 %) categories than in those in the reference category (61.0 %), although the differences were not significant (p=0.425 and 0.196, respectively).

**Table 5:** Prevalence of early tooth eruption (ETE) and odds ratios (ORs) and 95% confidence intervals (CIs) of ETE prevalence according to exposure during pregnancy and/or exposure after birth including the category of no exposure to both exposure types as reference

*Exposure type*	*ETE % (n/total n)*	*Crude*		*Adjusted*	
		*OR, 95% CI*	*p*	*OR, 95% CI*	*p*
**No exposure^a^**	61.0 (150/246)	Reference		Reference	
**During pregnancy only**	68.4 (13/19)	1.39, 0.51–3.77	0.522	1.54, 0.53–4.45	0.425
**After birth only**	79.3 (65/82)	2.45, 1.35–4.42	0.003	2.29, 1.19–4.40	0.013
**Both types of exposure^b^**	70.7 (29/41)	1.55, 0.75–3.18	0.235	1.71, 0.76–3.85	0.196

a No exposure to maternal smoking during pregnancy and household smoking after birth

b Exposure to maternal smoking during pregnancy and household smoking after birth

## DISCUSSION

ETE prevalence was greater by 16% points among children exposed to household smoking every day after birth than among those who were not exposed. The association was independent of potential confounders, including maternal smoking exposure during pregnancy, and a dose-response relationship was observed. Although the observed association is represented by canines in the study, these results suggest that children who are exposed to SHS after birth have a greater chance of contact between premature enamel crystals and acid produced by fermenting oral bacteria as the basic etiology of dental caries.

The association between ETE and SHS exposure during pregnancy was consistently insignificant, although ETE prevalence was greater by 11.4% points among children who were exposed to maternal smoking every day than among those who were not exposed. The trend in ETE prevalence was unexpectedly different with regard to the subgroups for SHS exposure during pregnancy (Table 4). Reasons for the lack of significance may include the inadequate sample size of this subgroup. AOR of ETE prevalence for the medium-dose exposure was less than one (Table 3). Furthermore, children whose mother ceased smoking partway during the pregnancy exhibited an even lower ETE prevalence than the reference (62.5% vs 65.5%, data not shown in the results section). A similar trend in the role of exposure timing was reported for growth after birth. Maternal smoking exposure in the second and third trimester predicted an accelerated conditional weight-for-length gain by 2 years of age^20^. The association between maternal smoking during the first trimester and dental eruption time was not significant^17^. Therefore, inadequate data regarding the timing of exposure during pregnancy may be added to the reasons for the lack of significance.

The role of gestational age in ETE based on chronological age may be a third reason for the lack of significance. Because this study focused on the role of ETE in the etiology of dental caries with respect to the timing of contact between the tooth surface and acid, gestational age was not available for analyses in this study. Delayed eruption with respect to chronological age is accounted for by premature birth (i.e. shortened gestation in children with very low birthweight)^21^, and may not be related to delayed dental development^22^. Because the causal association between maternal smoking and preterm delivery and shortened gestation was apparent^4^, the association between SHS exposure during pregnancy and ETE may be underestimated in children with a shorter gestational age. Furthermore, birth weight must be analyzed in relation to gestational age in weeks. Further studies that involve variables such as timing of exposure during pregnancy and gestational age are required to test the association between ETE and SHS exposure during pregnancy in detail.

A causal association of SHS exposure after birth with ETE should be carefully assessed in addition to the dose–response relationship, which was consistently significant for SHS exposure after birth. The biological plausibility is responsible for a causal inference. SHS exposure may influence the apoptosis of surrounding tissues in the alveolar bone adjacent to the coronary part of the erupting tooth^23^. Tobacco constituents enhance apoptosis in periodontal tissue^24-26^. Thus, the space generated by cell death allows the tooth to move toward the eruption site, resulting in ETE. Another theory is derived from the role of SHS exposure in catch-up growth or in the compensatory acceleration of growth rate^27^. This pathway is only applicable to the effect of exposure during pregnancy, whereas the association of a greater ETE prevalence with maternal smoking during pregnancy was not statistically significant.

Only small^16,18^ or no^17^ effects of SHS exposure during pregnancy on ETE were previously reported, wherein the association was adjusted for birth weight. Differences in ETE prevalence with regard to the exposure group were distinct in lower birth-weight (<3000 g) children, but the association between ETE prevalence and SHS exposure was not significant in children with a higher birth weight (≥3000 g). Therefore, stratification by birth weight would be an important strategy to assess the association between ETE and SHS exposure.

We did not use a criterion in accordance with the guidelines set in force by the WHO, for example, ≤2499 g corresponds to a low birth weight. This criterion was developed based on epidemiological findings regarding risk of infant death^28^. Children with low birth weight by this criterion are relatively few in Japan. The role of SHS exposure after birth on ETE in children with birth weight <3000 g may have impact on many children. Completion of primary teeth is likely correlated with body height and body mass^29^. Therefore, morphological measures may be considered for confounding variables to estimate the association between SHS exposure and timing of tooth eruption of children.

The strengths of this study are that children were surveyed at a particular age with two SHS exposure types, namely during pregnancy and after birth, and that children were stratified by birth weight. However, the interpretation of the association was beyond causal inference because of the cross-sectional design. Another limitation is that insufficient data regarding the dose were available to assess the dose-response relationship and the timing of exposure to assess the susceptible period of exposure for ETE to occur. Because the subjects in this study were not representative of Japanese children in the general population, the findings may not be generalized.

Exposure to nicotine may enhance the pathogenicity of cariogenic oral bacteria such as *Streptococcus mutans* ^11,30^. Unhealthy oral behaviors such as excessive sugar intake, poor oral hygiene, and infrequent dental visits are very common in children who are exposed to smoking^31^. Therefore, cariogenic contact between bacterially produced acid and the hard enamel of tooth surfaces may be facilitated by observed ETE and accelerated by SHS exposure after birth.

## CONCLUSIONS

Household smoking exposure after birth was independently associated with ETE of deciduous canines, and a dose-response relationship was observed. These results suggest that SHS exposure after birth predicts the increased probability of contact between premature enamel crystals of erupting deciduous teeth and acid produced by fermenting oral bacteria as the basic etiology of dental caries. Our findings warrant further study to examine whether ETE in children with SHS exposure after birth influences the incidence of early childhood caries.

## CONFLICTS OF INTEREST

Authors have completed and submitted the ICMJE Form for Disclosure of Potential Conflicts of Interest and none was reported.
