# Supplementary material for: Association between secondhand smoke exposure and early eruption of deciduous teeth: A cross-sectional study
Source: Tob Induc Dis. 2018 Feb 21;16:04. doi: 10.18332/tid/84892 (PMC6659493; doi:10.18332/tid/84892)
Supplement: Supplementary file 1 [file TID-16-04-s1.pdf]

Table 1. Association between ETE and potential confounders

**Categorical variable**	Variable	Category	Number (%) of ETE	Total	p
	Gender	Male	139 (70.2)	198(100)	0.092
		Female	118 (62.1)	190(100)	
	Residence	A city	38 (55.9)	68(100)	0.011
		B city	158 (72.5)	218(100)	
		C city	61 (59.8)	102(100)	
	Birth order	First	124 (68.9)	180(100)	0.304
		≥Second	133 (63.9)	208(100)	
	Highest education of parents	≤High school	82 (67.2)	122(100)	0.783
		≥College	175 (65.8)	266(100)	
	Annual income of parents, million yen	<3	35 (63.6)	55(100)	0.585
		3–4.9	132 (68.8)	192(100)	
		≥5	90 (63.8)	141(100)	
		Total	257 (66.2)	388(100)	
**Continuous variable**	Variable	ETE category	N	Mean±S.D.	
	Age, month	No	131	18.5±0.7	0.001
		Yes	257	18.8±0.9	
		Total	388	18.7±0.8	
	Birth weight, g	No	131	2912.9±350.5	0.020
		Yes	257	3013.7±424.7	
		Total	388	2979.7±403.6	

Table 2. Prevalence of early tooth eruption (ETE) and odds ratios (ORs) and 95% confidence intervals (CIs) of ETE according to SHS exposure level with regard to exposure type in children with birth weights of ≥3000 g

**Exposure type**	Exposure level	ETE	Crude		Adjusted	
		% (n/total n)	OR, 95% CI	p	OR, 95% CI	p
**Maternal smoking during pregnancy**	No exposure	73.3 (118/161)	Reference		Reference	
	Medium dose	66.7 (16/24)	0.73, 0.29-1.83	0.499	0.62, 0.23-1.67	0.343
	Highest dose	100 (2/2)	Not available		Not available	
			p for trend = 0.698		p for trend = 0.841	
**Household smoking after birth**	No exposure	70.2 (85/121)	Reference		Reference	
	Medium dose	80.0 (24/30)	1.69, 0.64-4.50	0.207	2.02, 0.68-5.99	0.207
	Highest dose	75.0 (27/36)	1.27, 0.54-2.97	0.534	1.35, 0.53-3.42	0.543
			p for trend = 0.365		p for trend = 0.377	
	Total	72.7 (136/187)				

Published by EUEP European Publishing on behalf of the International Society for the Prevention of Tobacco Induced Diseases (ISPTID)
© 2018. Hanioka T. This is an Open Access article distributed under the terms of the Creative Commons Attribution 4.0 International License.
(https://creativecommons.org/licenses/by/4.0/)
